# Effect of continuous use of metformin on kidney function in diabetes patients with acute myocardial infarction undergoing primary percutaneous coronary intervention

**DOI:** 10.1186/s12872-020-01474-5

**Published:** 2020-04-21

**Authors:** Qi Yu, Jia-Jia Zhu, Wen-Xian Liu

**Affiliations:** Department of Cardiology, Beijing Anzhen Hospital, Capital Medical University, Beijing, 100029 China

**Keywords:** Contrast induced-acute kidney injury, Metformin, ST-segment elevation myocardial infarction

## Abstract

**Background:**

Diabetes patients presenting with ST-segment elevation myocardial infarction (STEMI) scheduled for primary percutaneous coronary intervention (PCI) have an increased risk of contrast induced-acute kidney injury (CI-AKI). The effects of continuous use of metformin on kidney function are still controversial in patients submitted to primary PCI. This study aimed to assess continuous metformin therapy on kidney function in diabetic patients undergoing coronary intervention.

**Methods:**

Two hundred eighty-four patients with metformin-treated diabetes, who underwent coronary intervention within 24 h for STEMI, were enrolled in the retrospective study. All the patients had estimated glomerular filtration rate (eGFR) of > 30 mL/min/1.73 m^2^. According to the physicians’ decisions after admission, 119 patients continued metformin treatment after primary PCI, while 165 patients discontinued it > 48 h after the procedure. Serum creatinine was collected at admission and within 48 h post primary PCI to evaluate the incidence of CI-AKI. We performed a multiple logistic regression analysis to examine the determinants of CI-AKI.

**Results:**

No statistical difference in CI-AKI incidence between the continuous and the discontinuous metformin group (12.6%vs10.3%, *p* = 0.545). Multivariable logistic regression analysis indicated eGFR ≤60 ml/min/1.73 m^2^[*p* = 0.025, OR: 3.131; 95% CI (1.156–8.482)] and contrast volume [*p* = 0.002, OR: 1.010; 95% CI (1.004–1.016)] were predictive factors of CI-AKI. Metformin therapy was irrelevant to CI-AKI [*p* = 0.365, OR: 0.698; 95% CI (0.320–1.521)]. No case of lactic acidosis was found in this study. Besides, the study supported discontinuation of metformin was not beneficial for patients’ blood glucose control after admission.

**Conclusions:**

The study indicated that the metformin continuation after primary PCI for STEMI in diabetic patients with eGFR > 30 ml/min / 1.73 m^2^ did not increase the risk of CI-AKI.

## Background

As the first-line therapy of type 2 diabetes mellitus (T2DM), metformin is applying to nearly one-third of diabetes patients worldwide [1]. Therefore, in urgent situations, such as acute myocardial infarction, many of them cannot withhold the metformin prior to percutaneous coronary intervention (PCI)-related contrast medium administration [2]. Cleared by the kidneys, metformin is accumulated in circumstances when kidney function is deteriorated, such as contrast-induced acute kidney injury (CI-AKI), which may lead to lactic acid accumulation. Although metformin associated with lactic acidosis (MALA) has a reported mortality of 30–50%, it is a rare disease with an estimated incidence of 1–5 cases per hundred thousand people [3]. However, previous studies have shown that in most cases, metformin therapy may be merely concomitant and may not have a causal role at all [4].

ST-elevation myocardial infarction (STEMI) patients with CI-AKI after coronary stent implantation had longer hospital stays, increased adverse cardiovascular outcomes, and higher mortality [2]. As the direct toxicity of contrast media, the high thrombogenic state, inflammation, and the decrease in renal perfusion, patients submitted to primary PCI for STEMI are at high-risks for CI-AKI, [5]. According to different definition, the incidence of CI-AKI can range from approximately 6.4 to 27.7% in STEMI patients after primary PCI [6].

Because of the post-procedural risk-MALA and CI-AKI in diabetic patients submitted to PCI for STEMI, the guidelines are inconsistent on whether to continue metformin in patients undergoing PCI [7–10]. Currently, studies had concluded that initiation of metformin ≤ 3 h after primary PCI and chronic metformin therapy before primary PCI had no influence on CI-AKI in STEMI patients [2, 11]. However, there is no clinical data about the continuation of metformin treatment during the primary PCI period in diabetic patients with STEMI, and the effects of metformin on the kidneys are still widely debated in patients exposed to contrast agents [12, 13].

The objective of the study to was to evaluate the influence of continuous metformin therapy on kidney function after coronary intervention for STEMI. In the meantime, the independent predictors of CI-AKI were explored.

## Methods

### Patients and study design

A single-centre retrospective study in a cohort of all-comers with T2DM undergoing primary PCI for STEMI was performed between January 2008 and December 2018 in the Department of Cardiology of Beijing Anzhen Hospital. All patients received metformin therapy before PCI. STEMI and T2DM are defined depending on the current guidelines [8, 14]. The exclusion criteria included: patients without PCI, deficient of creatinine data, end-stage kidney disease (estimated glomerular filtration rate < 30 ml/min/1.73 m^2^), respiratory failure, severe infections, liver disease, a history of alcoholism, cardiogenic shock, and death ≤48 h after hospitalization. A total of 284 consecutive T2DM patients presenting with STEMI, showed an onset of symptoms < 12 h were enrolled in the study. Whether the patient stopped taking the metformin during the peri-angiography period depended on the decisions of the physicians at the time. Patients in group 1 continued to take metformin normally while those in group 2 stopped metformin upon admission and restarted it > 48 h after PCI. Non-ionic, low or equal osmolality contrast agent was used in all patients chosen by each doctor. All patients signed informed consent before enrolled in the study. The study protocol was approved by the institutional ethics committee of Beijing Anzhen Hospital, Capital Medical University.

### Data collection

Data collection regarding cardiovascular risk factors, clinical variables, medication prescriptions and, laboratory values were conducted based on a review of medical records. Cardiovascular risk factors included age, gender, diabetes duration, hyperlipidemia, current smoking, hypertension, etc. Clinical variables included contrast media volume, time to PCI, single-vessel disease, infarct-related artery, etc. The daily metformin dose was recorded and categorized as > 0 g to ≤0.5 g, > 0.5 g to ≤1 g, > 1g to ≤1.5 g, or > 1.5 g. The medication prescriptions initiated during hospitalization were recorded. Laboratory values, including HbA1c (glycated haemoglobin), Hb (haemoglobin), cTnI (cardiac troponin I), and creatinine, were collected at admission and after PCI.

### Primary outcome

The study’s primary outcome was CI-AKI. The methods for calculating the estimated glomerular filtration rate (eGFR) and incidence of CI-AKI have been described in other articles and are briefly summarized below [2]. The serum creatinine concentration was measured in all patients upon hospital admission and daily in the early 2 days after intracoronary stenting. The highest serum creatinine level within 2 days after coronary angiography was used as post PCI creatinine to diagnose CI-AKI. The diagnostic criteria of CI-AKI was an elevation in serum creatinine ≥ 27 μmol/l or ≥ 50 per-cent over baseline within 48 h after contrast media injection [5]. Then the association between continuous metformin therapy in the perioperative period and incidences of CI-AKI was examined.

### Secondary outcomes

The secondary outcomes were (i) the peak blood glucose and insulin initiation therapy within 2 days after primary PCI and (ii) lactic acidosis occurred during hospitalization. Capillary glucose was measured at least four times a day during the hospital stay, including once fasting blood glucose and three times postprandial blood glucose, and the peak values within 48 h after primary PCI for each variable were considered. The peak values of fasting and postprandial blood glucose within 48 h after primary PCI were compared between the two groups of patients without insulin initiation therapy. Patients with initiated insulin therapy within 48 h after surgery were recorded, either initiating subcutaneous insulin injection or continuous intravenous insulin. Lactic acidosis was defined as a plasma lactate level of more than 5 mmol/L and arterial pH less than 7.35 [2].

### Statistical analysis

All statistical studies were carried out using the SPSS 23.0 program (Chicago, IL, USA). Categorical data were reported as percentages, and continuous data were reported as median (25 – 75th percentile) or mean (± SD). The distribution properties of the data were performed using the Kolmogorov-Smirnov test. Dichotomized data were analyzed for the significant difference using Chi-2 or Fisher’s exact test appropriately. Continuous data were analyzed for the significant difference using Student’s t or Mann–Whitney test appropriately. Finally, we identified the predictive factors of CI-AKI using logistic regression analysis.

## Results

### Baseline characteristics

Baseline characteristics and patient subgroups, depending on the discontinuation of metformin therapy are summarized in Table 1. According to the physicians’ decisions after admission, a total of 119 patients (group 1) did not stop metformin treatment during the peri-angiography period, and the other 165 patients (group 2) stopped metformin from upon admission and restarted > 48 h after PCI. There was no statistical difference in both groups for metformin dose. Patients continuing metformin therapy used more beta-blocker. Group 1 patients had cardiovascular risk factors similar to group 2. Clinical factors, including single-vessel disease and infarct-related artery, were also similar for both groups. Delays of management (time to PCI), left ventricular systolic function, cTnI peak, and glycosylated haemoglobin (HbA1c) values were comparable between the two groups (Table 2).

**Table 1:** Cardiovascular risk factors, clinical data and medication (n (%) or median [IQR]) (*n* = 284)

metformin	Metformin *n* = 119	No Metformin *n* = 165	*p* value
Risk factors			
Age ≥ 65 years	28 (23.5%)	46 (27.9%)	0.410
Women	26 (21.8%)	47 (28.5%)	0.207
Diabetes duration≥5 years	80 (67.2%)	99 (60.0%)	0.430
Hypertension	74 (62.2%)	114 (69.1%)	0.225
Hyperlipidemia	53 (44.5%)	61 (37.0%)	0.199
Current smoking	80 (67.2%)	99 (60.0%)	0.213
Stroke	8 (6.7%)	14 (8.5%)	0.586
Prior myocardial infarction	16 (13.4%)	12 (7.3%)	0.085
PAD	3 (2.5%)	4 (2.4%)	0.959
Medical therapy initiated during hospitalization			
Other oral antihyperglycemic agent.	60 (50.4%)	73 (44.2%)	0.303
ACEI or ARB	51 (42.9%)	68 (41.2%)	0.782
Mineralocorticoid receptor antagonist	4 (3.4%)	7 (4.2%)	0.946
Calcium-channel blocker	6 (5.0%)	5 (3.0%)	0.579
Beta-blocker	95 (79.8%)	99 (60%)	< 0.05
Metformin dosage			0.230
> 0 g to ≤0.5 g	1 (0.8%)	7 (4.2%)	
> 0.5 g to ≤1 g	32 (26.9%)	36 (21.8%)	
> 1 g to ≤1.5 g	85 (71.4%)	118 (71.5%)	
> 1.5 g	1 (0.8%)	4 (2.4%)	
Clinical data			
LVEF≤40%	7 (5.9%)	11 (6.7%)	0.789
Time to PCI (hour)	4 (6–16)	6 (4–9)	0.139
Single--vessel disease	31 (26.1%)	45 (27.3%)	0.818
Infarct-related artery			
Left main	0 (0%)	1 (0.6%)	1.000
Left anterior descending coronary artery	56 (47.1%)	83 (50.3%)	0.589
Left circumflex coronary artery	17 (14.3%)	20 (12.1%)	0.593
Right coronary artery	47 (39.5%)	60 (36.4%)	0.591
Contrast media volume (ml)	120 (120–200)	150 (110–200)	0.884

*PAD* peripheral arterial disease, *ACEI* angiotensin-converting enzyme inhibitor, *ARB* angiotensin receptor blocker, *LVEF* left ventricular ejection fraction, *PCI* percutaneous coronary intervention

**Table 2:** Laboratory data and incidence of CI-AKI (n (%) or median [IQR]) (*n* = 284)

	metformin *n* = 119	No Metformin *n* = 165	*p* value
HbA1c(%)	7.0 [3.6–14.2]	7.8 [6.9–8.8]	0.194
Hb(g/l)	141 [126–152]	120 [110–140]	0.079
cTnI peak (ng/ml)	7.0 [3.6–14.2]	7.8 [6.9–8.8]	0.842
Creatinine			
Baseline (μmol/l)	76 [66–86]	73 [61–84]	0.105
Post PCI (μmol/l)	83 [72–98]	76 [64–76]	0.012
Relative creatinine change	11% [6–20%]	11% [5–22%]	0.858
post PCI (%)			
≥ 50% increase	5 (4.2%)	9 (5.5%)	0.630
Absolute creatinine change	7.9 [2.4–13.2]	6.7 [0.2–12.7]	0.198
post PCI (%) (μmol/l)			
≥ 27 μmol/l(0.3 mg/dl)	15 (12.6%)	15 (9.1%)	0.342
eGFR (ml/min/1.73m^2^)			
Baseline (ml/min/1.73m^2^)	89 [73–104]	94 [72–113]	0.170
eGFR≤60 ml/min/1.73m^2^	9 (7.6%)	21 (12.7%)	0.163
Post PCI (ml/min/1.73m^2^)	77 [65–95]	87 [67–102]	0.092
Relative eGFR change	13.4% [6.4–25.1%]	13.5% [5.2–24.3%]	0.449
post PCI (%)			
Absolute eGFR change	−7 [−16; 3]	−6 [−18; 3]	0.981
post PCI (ml/min/1.73m^2^)			
CI-AKI	15 (12.6%)	17 (10.3%)	0.545

*HbA1c* glycosylated hemoglobin, *Hb* hemoglobin, *eGFR* glycosylated hemoglobin estimated glomerular filtration rate, *cTnI* cardiac troponin I, *PCI* percutaneous coronary intervention, *CI-AKI* contrast induced-acute renal injury

### Kidney function

We found both baseline eGFR and postoperative eGFR were comparable between the two groups (*p* = 0.170 and *p* = 0.092, respectively) (Table 2). Besides, the relative and absolute creatinine changes in patients who continuously used metformin were comparable to those who discontinued metformin after PCI (*p* = 0.858 and *p* = 0.198, respectively). And it was found that both groups had a similar increase of about 7% in Cr values. Furthermore, no statistical difference was shown in the incidence rate of CI-AKI between the two groups (12.6% VS 10.3%, *p* = 0.545).

### Blood glucose control

Group 2 patients who discontinued metformin treatment showed the higher rate of initiating insulin therapy ≤48 h after primary PCI, whether initiating subcutaneous insulin injection or continuous intravenous insulin (*p* = 0.001 and *p* = 0.021, respectively) (Table 3). In the subgroup of the 207 patients who did not initiate insulin therapy ≤48 h after primary PCI, 102 (49%) continued the metformin therapy. Both fasting and postprandial glucose peaks were significantly higher in patients (51%) discontinued metformin (7.75[7.10–9.95] VS 9.10[7.30–11.42], 10.65[9.00–12.20] VS 13.85[11.90–16.20], respectively) (Table 4).

**Table 3:** Initiate Insulin therapy ≤48 h after primary PCI

	metformin *n* = 119	No Metformin *n* = 165	*p* value
initiate continuous intravenous insulin	2 (1.68%)	13 (7.88%)	0.021
Initiate subcutaneous insulin injection	15 (12.61%)	47 (28.48%)	0.001

**Table 4:** Blood glucose control ≤48 h after primary PCI (excluded patients initiating insulin therapy ≤48 h after primary PCI)

	metformin	No Metformin	*p* value
	*n* = 102	*n* = 105	
fasting glucose peak (mmol/l)	7.75 [7.10–9.95]	9.10 [7.30–11.42]	< 0.05
postprandial glucose peak (mmol/l)	10.65 [9.00–12.20]	13.85 [11.90–16.20]	0.02

**Table 5:** Single-factor and multiple-factor regression analysis of CI-AKI

Factor	Single-factor		Multiple-factor	
	OR (95% CI)	p	OR (95%CI)	p
Metformin	0.796 (0.381,1.666)	0.546	0.698 (0.320,1.521)	0.365
Metformin dose	0.787 (0.413,1.500)	0.467	0.658 (0.336,1.288)	0.222
eGFR≤60 ml/min/1.73m^2^	2.788 (1.087,7.147)	0.033	3.131 (1.156,8.482)	0.025
Contrast media volume (ml)	1.009 (1.004,1.015)	0.002	1.010 (1.004,1.016)	0.002

*CI-AKI* contrast induced-acute renal injury, *eGFR* glycosylated hemoglobin estimated glomerular filtration rate

### Multiple regression analysis for CI-AKI

Univariate analysis showed that metformin was irrelevant with the increased incidence of CI-AKI after contrast agent exposure. By using multiple regression analysis, we included variables which were significant in univariate analysis, found the presence of contrast volume (*p* = 0.002) and eGFR < 60 ml/min/1.73 m^2^ (*p* = 0.025) were associated with CI-AKI (Table 5).

## Discussion

In this study, the continuous use of metformin for T2DM treatment in STEMI patients, who were submitted to primary PCI, was found reliable concerning the development of CI-AKI. It was also found eGFR ≤ 60 ml/min/1.73 m^2^ and the contrast volume were risk factors of CI-AKI. Besides, discontinuation of metformin results in interruption of hypoglycemic treatment or change of hypoglycemic regimen, and patients presented glucose fluctuation after admission. It was the first study to demonstrate that diabetic patients continuing metformin treatment during primary PCI period were not correlated with CI-AKI.

T2DM is correlated with poor prognosis in patients who have non-obstructive or obstructive stable coronary artery disease. Recent studies demonstrated that diabetes increase mortality and adverse cardiac outcomes in patients with non-obstructive coronary artery stenosis (NOCS)-acute myocardial infarction (AMI) [15, 16]. Diabetes may favour the plaque instability in the context of NOCS through pathogenetic mechanisms including inflammation, endothelial dysfunction and coronary vasospasm [15]. As a first-line drug for T2DM, metformin has been shown to have cardiovascular benefits and fewer adverse reactions [17]. Current guidelines suggest that prediabetes should also be treated with metformin to mitigate the risk of developing diabetes. A recent study by Celestino et al. found that metformin therapy may improve adverse cardiovascular outcomes in prediabetes patients by reducing coronary endothelial dysfunction. The improvement in endothelial dysfunction is attributed to metformin downregulating the inflammation/ oxidative stress in the context of NOCS [18]. Besides, metformin therapy for prediabetes may improve outcomes by a reduction of inflammatory tone and leptin to adiponectin rate in peri-coronary fat in AMI patients [19]. There is increasing evidence that specific hypoglycemic drugs with pleiotropic effect on inflammatory tone and oxidative stress may affect the control of atherosclerotic plaque progression in AMI patients [15].

Patients presenting with STEMI are prone to develop CI-AKI after primary PCI, and high thrombogenic state and inflammation are among the leading causes [5]. Hyperglycemic patients presenting with STEMI have higher coronary thrombus burden compared with thrombi from normoglycemic counterparts. Evidence showed that hyperglycemia causes overproduction of reactive oxygen species and inflammation from thrombus plaque, favouring thrombotic embolization and poor myocardial infarction outcomes. The miR33/ sirtuin 1 pathway have been demonstrated to play a part in promoting inflammatory and coagulation of coronary thrombi in STEMI patients during hyperglycaemia [20, 21]. In this context, whether metformin in diabetic patients with STEMI undergoing coronary angiography should be discontinued, has been discussed because of its post-procedural risks, including CI-AKI and MALA. Advice on the discontinuation of metformin differs between guidelines [7–10, 22]. European Society of Urogenital Radiology guideline recommends stopping metformin directly from the time of contrast media administration [10], while European Society of Cardiology guidelines recommends checking renal function after angiography for at least 3 days and withhold metformin when renal function is deteriorated [8]. There are few studies about metformin used in patients with mildly impaired kidney function after the administration of contrast agents. In the two latest randomized controlled studies, the patients continuing metformin during peri-angiography does not carry the excess risk for renal dysfunction. No lactic acidosis is observed in both studies [23, 24]. Currently, the influence of metformin on the kidney function in STEMI patients is widely discussed. The GIPS III trial has supported the idea that metformin is safe to use after STEMI and contrast agent exposure. Non-diabetic patients started metformin therapy within 3 h after the coronary intervention had no harmful effect on kidney functions [11]. In addition, a multi-centre observational study has shown that chronic metformin treatment before PCI has no significant effect on CI-AKI in T2DM patients with STEMI [2]. Those studies strongly suggested that metformin is not related to an increased risk of renal dysfunction after coronary angiography. The hypothesis that AMI patients may use metformin safely during the peri-angiography period was further reinforced. Our research is consistent with the above studies. The present data indicated that both absolute and relative creatinine change after PCI were similar between the patients continuing metformin therapy and those suspending metformin therapy. By multivariate analysis, metformin was not related to CI-AKI, whereas contrast volume (*p* = 0.002) and eGFR < 60 ml/min/1.73 m^2^ (*p* < 0.025) were indicated to be predictive factors of CI-AKI.

Several studies have demonstrated that the contrast agent volume is associated with the morbidity of acute kidney injury [25]. The nephrotoxicity of iodinated contrast media may be proportional to the dose for coronary angiography. The main causes of CI-AKI have been proposed, including renal medullary hypoxia caused by hemodynamic instability, oxidative stress and direct toxicity on kidney tubular epithelial cells [26–28]. It was important to understand that using a lower dose of contrast agent may substantially reduce the CI-AKI risk of patients. Patients with chronic kidney disease have fewer nephron units than normal, so exposure to the same volume of contrast media will significant increased proportionally. Because of their low adaptive capacity and increased contrast agent exposure, they are more susceptible to develop CI-AKI [27].

As metformin is eliminated by the kidneys, there are concerns that in patients with the reduced kidney function, the lactic acidosis will be accumulated and precipitated [29]. However, the strength of the relationship between metformin and lactic acidosis has been dramatically overstated [4]. Several clinical studies have shown that there is no significant correlation between metformin concentration and lactic acidosis [1, 4, 30–32]. Furthermore, the current study and meta-analyses show that the morbidity of lactic acidosis using metformin is not significantly different from other hypoglycemic treatments, such as sulfonylureas, insulin, and other oral hypoglycemic agents [33–39]. Other studies have also shown that metformin concentrations remain in a therapeutic range in mildly to moderately renal impaired patients [40]. At the same time, growing evidence suggests that the underlying disease associated with the tissue hypoxia rather than metformin use is related to lactic acidosis in diabetes [41–43]. In the present research, no case of lactic acidosis was observed during hospitalization for both groups.

Hyperglycemia has been linked with more complications during hospitalization and poor outcomes in AMI patients. Lazzeri et al. [44] concluded that in STEMI patients, in-hospital peak glycemia is negatively correlated with long-term survival. Besides that, another study had also confirmed that acute hyperglycemia is a predictor of CI-AKI and in-hospital mortality [45]. Peri-procedural tight glycemic control has been shown to significantly increase the area of myocardial salvage following a great recovery of left ventricular function in hyperglycemic patients undergoing emergency coronary intervention for STEMI. These observations strongly suggest that the tight glycemic control at the time of the PCI may be pursued in the STEMI patients to improve their prognosis [46]. Therefore, strict glucose management in STEMI patients with mildly impaired renal function is recommended during a hospital stay [47]. In this research, we found the patients who discontinued metformin treatment, were more likely to initiate insulin therapy and had higher peak glycemia. Our data indicated that the patients who stopped metformin were inclined to have blood glucose fluctuation and changes in hypoglycemic regimens after admission.

Unlike previous studies, our study focused on the effect of perioperative use of metformin on renal function, with one group continued metformin treatment after primary PCI and another discontinued it > 48 h after the primary PCI, which is different from Zeller’s [2] work, and this is a major contribution of our work. The application of metformin in the peri-angiography period in the existing guidelines is not yet consistent; our study focused on this situation and target at patients who are inclined to develop CI-AKI. The result of the study indicated that continued use of metformin did not impair renal function compared to discontinuation of metformin during primary PCI period. In real clinical practice, we found that discontinuous use of metformin caused problems in blood glucose management in STEMI patients after admission. The patients who stopped metformin were more likely to have blood glucose fluctuation and changes in hypoglycemic regimens after PCI. The peak values of fasting and postprandial blood glucose in patients with discontinuous metformin treatment were significantly higher than those of patients receiving metformin continuously. Previous studies have confirmed that peak glycemia during hospitalization is negatively correlated with the long-term survival in diabetic STEMI patients [39]. To summarize the above findings, continuous use of metformin during coronary angiography may not raise the risk of CI-AKI, and the blood glucose of patients after admission will be better controlled, which is conducive to the prognosis of STEMI patients.

This study had the following two limitations. Firstly, it was a retrospective cohort study, conducted at a single centre, based on a relatively small size of populations. Secondly, since the high-risk patients such as those who needed intra-aortic balloon pump, or had respiratory failure were excluded, the results in this paper may not be adapted to these subgroups of patients. However, it was believed that the findings are of clinical significance in most patients.

## Conclusions

The present results indicated that the continuous use of metformin after PCI for STEMI in diabetic patients with GFR > 30 ml/min / 1.73 m^2^ may not enhance the risk of CI-AKI. Upon multivariable adjustment, eGFR < 60 ml/min/1.73 m^2^ and contrast agent dose were linked with CI-AKI after primary PCI, while the continuous metformin treatment was not. Besides that, the patients who discontinued metformin treatment showed an increased rate of initiating insulin therapy and higher peak glycemia within 48 h after primary PCI.

## Data Availability

The datasets during and/or analysed during the current study available from the corresponding author on reasonable request.

## Abbreviations

STEMI: ST-segment elevation myocardial infarction
PCI: Percutaneous coronary intervention
CI-AKI: Contrast induced-acute kidney injury
eGFR: Estimated glomerular filtration rate
T2DM: Type 2 diabetes mellitus
MALA: Metformin associated with lactic acidosis
PAD: Peripheral arterial disease
LVEF: Left ventricular ejection fraction
ACEI: Angiotensin-converting enzyme inhibitor
ARB: Angiotensin receptor blockers
HbA1c: Glycated haemoglobin
Hb: Haemoglobin
cTnI: Cardiac troponin I
NOCS: Non-obstructive coronary artery stenosis
AMI: Acute myocardial infarction
